# The Influence of Stent-Strut Morphology on Iliac Limb Hemodynamics During EVAR in Compliant 3D-Printed Arterial Models

**DOI:** 10.3390/jcm15103768

**Published:** 2026-05-14

**Authors:** Maciej Wojtuń, Arkadiusz Kazimierczak, Miłosz Kawa, Aleksander Falkowski, Piotr Gutowski, Patryk Skórka, Paweł Rynio

**Affiliations:** 1 Department of General, Dental and Interventional Radiology, Pomeranian Medical University in Szczecin, Al. Powstańców Wielkopolskich 72, 70-111 Szczecin, Poland; wojtunmaciek@gmail.com (M.W.);; 2 Department of Vascular Surgery and Angiology, Pomeranian Medical University in Szczecin, Al. Powstańców Wielkopolskich 72, 70-111 Szczecin, Poland

**Keywords:** EVAR, iliac artery angulation, tortuosity, stent-graft, stent-strut morphology, 3D printing, vascular phantom

## Abstract

**Background:** Endovascular aortic aneurysm repair (EVAR) is considered the gold standard for the treatment of abdominal aortic aneurysms. However, the performance of stent-grafts used during this procedure may be affected by their structural design, particularly in anatomically challenging, tortuous iliac arteries. This study aimed to evaluate the hemodynamic performance of different stent-graft limb designs in an in vitro EVAR simulation using compliant three-dimensional (3D)-printed iliac artery models with controlled angulations. **Methods:** Four commercially available stent-grafts (Anaconda^®^, Endurant II^®^, Treo^®^, Zenith Spiral-Z^®^) representing different stent-strut configurations (including O-ring, Z-stent, and spiral designs) were deployed in compliant 3D-printed vascular phantoms simulating severe iliac angulations of 75°, 90°, and 105°. The models were incorporated into a pulsatile flow circuit, and pressure and flow velocity were measured proximally and distally to the angulated segment. **Results:** Across all tested angulations, the O-ring-based design demonstrated the most favorable hemodynamic performance. In particular, the Anaconda stent-graft showed the smallest pressure loss and the lowest increase in distal flow velocity, especially in the 90° and 105° models. These findings suggest that O-ring-supported structures provide greater flexibility and conformability in severely angulated iliac segments. **Conclusions:** In this controlled in vitro setting, stent-grafts with O-ring strut morphology better preserved flow conditions than other tested configurations in tortuous anatomy. These results suggest that stent-graft structural design may influence device behavior in challenging iliac anatomy under controlled in vitro conditions. These findings should be considered hypothesis-generating bench data and do not represent direct evidence for clinical device selection.

## 1. Introduction

An abdominal aortic aneurysm (AAA) is a life-threatening abnormal dilation of the abdominal aorta to at least 1.5 times its physiological diameter [1,2]. The aim of AAA treatment is to prevent fatal aneurysm rupture associated with high mortality. Previously, we determined differences in wall structure between ruptured and unruptured aneurysms [1,2]. Endovascular aneurysm repair (EVAR) is a minimally invasive procedure characterized by reduced mortality and morbidity, owing to lower early complication rates, less trauma, and shorter hospitalization periods compared with traditional open aortic surgery. For AAA, EVAR is currently the preferred treatment option [3,4].

The success of EVAR depends largely on the anatomical conditions of the vascular system. Proper fixation and sealing of the stent-graft require adequate landing zones and appropriate vessel geometry [4,5,6,7]. Anatomical limitations include the length, diameter, and angulation of the aneurysm neck, as well as the diameter, length, angulation, bends, kinking, or tortuosity of the iliac arteries [4]. In clinical practice, severely angulated or tortuous iliac arteries may impair stent-graft conformability, potentially leading to graft deformation, disturbed flow, or limb occlusion during follow-up.

Several commercial EVAR devices differ in their construction and stent-strut configuration [8]. Stent-graft limbs may be supported by Z-stents, M-stents, O-rings, or spiral structures, which influence their flexibility and adaptation to curved vascular anatomy [9,10,11]. Some configurations may better preserve lumen geometry in angulated vessels, whereas others may be more prone to collapse or lumen narrowing, potentially affecting local hemodynamics and long-term patency [3,12,13,14,15]. However, the performance of different stent-strut designs in severely angulated iliac segments remains insufficiently characterized.

Experimental in vitro simulations using compliant three-dimensional (3D) printed vascular phantoms enabled controlled evaluation of device behavior under defined geometric conditions. Such models enable the assessment of hemodynamic parameters while isolating the influence of vessel angulation and device structure.

**Figure 1:**
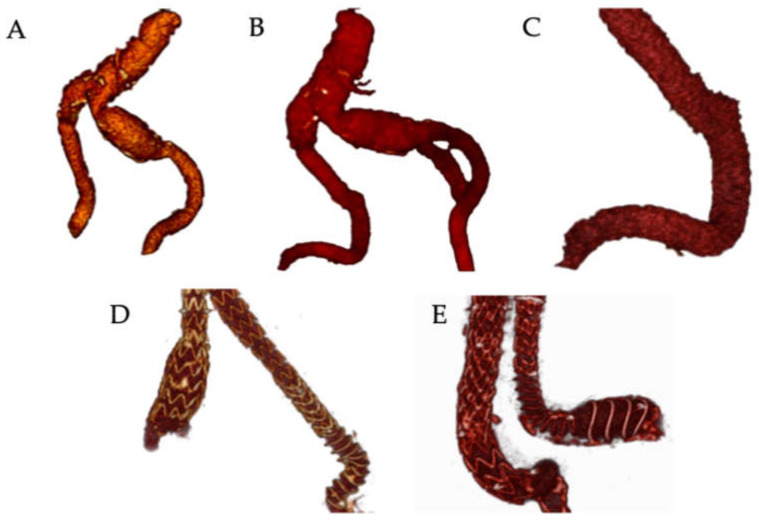
Computed Tomography Angiography (CTA) reconstructions of angulated iliac arteries before and after stent-graft implantation. (**A**,**B**); CTA angulated iliac arteries before implantation of stent-graft; (**C**) CTA angulated left iliac and external iliac artery before implantation of stent-graft; (**D**) CTA angulated iliac arteries after stent-graft implantation (Endurant II and Anaconda—angulated left EIA); (**E**) CTA angulated iliac arteries after stent-graft implantation (Endurant II—angulated right CIA and Anaconda—angulated left CIA/EIA). CIA—common iliac artery; EIA—external iliac artery.

Therefore, the aim of this study was to evaluate the influence of different stent-strut configurations on the hemodynamic performance of commercially available stent-graft limbs deployed in compliant 3D-printed iliac bend phantoms with controlled angulations (Figure 1).

## 2. Materials and Methods

### 2.1. Manufacturing of Arterial Models with Angulation

**Figure 2:**
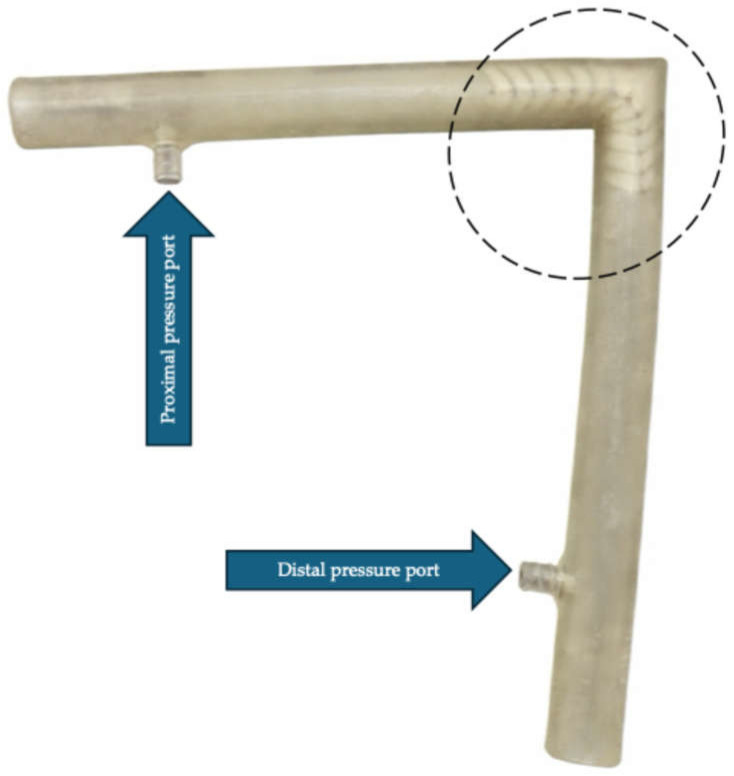
Representative 3D-printed phantom of a severely angulated arterial segment with an implanted stent-graft (dashed circle) used for fluid-dynamic simulation of physiological arterial flow. Proximal and distal pressure measurement connectors (arrows) are indicated.

To understand the hemodynamic conditions and biomechanical forces generated by vascular kinking, it was necessary to use compliant arterial models that approximate in vivo conditions. For this purpose, we employed standardized 3D-printed models of iliac arteries with controlled angulations, with different stent-grafts implanted in situ. The 3D models of iliac arteries were designed using Autodesk Fusion 360 (Autodesk Inc., San Rafael, CA, USA) (Figure 2). The model length was 10 cm from each end to the angulated segment.

The flow lumen had a diameter of 11 mm with bends of 75°, 90°, and 105°. The 3D models had Luer-lock connectors at both ends to allow pressure measurement (the connectors were placed approximately 2 cm from the ends of the models). The compliant vessel setting was used to print the models on a J850 Digital Anatomy Printer (Stratasys, Eden Prairie, MN, USA). These steps were performed at the hospital’s 3D-printing medical technology laboratory by staff experienced in generating medical 3D models. The elastic compliant tubes were made from Agilus30^TM^ resin (Stratasys, Eden Prairie, MN, USA).

### 2.2. Flow Mock System

Stent grafts from four different manufacturers were implanted into the compliant 3D-printed models simulating severe iliac artery angulation. The tested stent grafts were Anaconda (Vascutek, Terumo, Inchinnan, UK), Zenith Spiral-Z (Cook, Limerick, Ireland), Endurant IIs (Medtronic, Dublin, Ireland), and Treo (Terumo Shibuya, Tokyo, Japan). Limbs of 13 mm diameter were used for all stent grafts except Anaconda, for which only a 12 mm diameter option was available. The nominal limb lengths were 80 mm for Anaconda, 82 mm for Endurant II, 80 mm for Treo, and 90 mm for Zenith Spiral-Z. Differences in device diameter and limb length represent a potential confounding factor in the hemodynamic comparison. In vitro, hemodynamics were replicated in the lumen of the 3D models using a Harvard Apparatus pump (Holliston, MA, USA). Flow pressure before and after the stent-graft limbs was measured using pressure sensors (Trandomed, Ningbo, China). Pump parameters were adjusted to deliver a stroke volume of 15 mL at a rate of 60 beats per minute, with a systolic duration of 0.33 s and a diastolic duration of 0.67 s. The flow loop included a fluid reservoir and connecting tubing, forming a closed circulation system that allowed stable pulsatile flow conditions during measurements. Pressure sensors were positioned approximately 2 cm proximally and distally to the angulated segment. Each stent-graft deployment and measurement was repeated five times for each angulation model. The pressure drop ratio was calculated for each stent-graft and angulation model and is summarized in Table 1.

**Figure 3:**
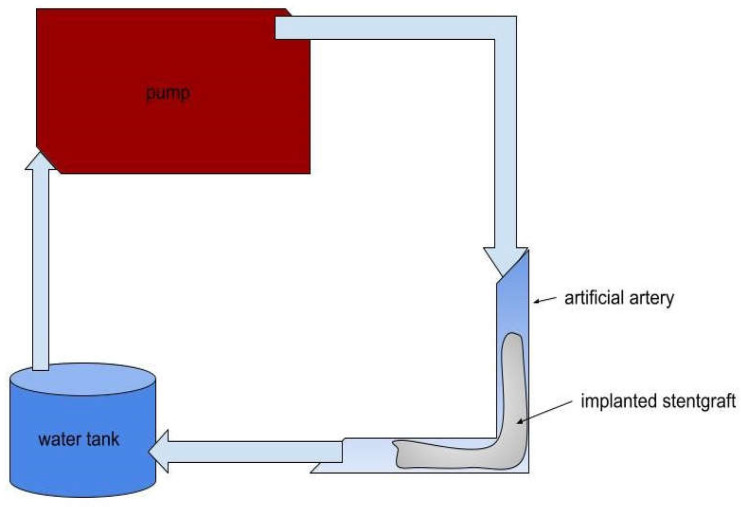
Schematic representation of the flow circulatory system using a computer-controlled gear pump to provide the fluid dynamic simulation of physiological flow through the 3D-printed kinked segment of iliac artery implanted with a selected stent-graft at varying angles. The arrows indicate the direction of fluid flow and identify the main components of the system.

Figure 3 shows the bench model of the hemodynamic system used to test the stent-grafts implanted into the angulated iliac artery models. For the blood analog, a 60:40 water-glycerol mixture was used. The fluid was heated to approximately 36 °C before perfusion, while measurements were performed at room temperature.

### 2.3. Doppler Ultrasound Technique

Doppler Ultrasound measurements of flow velocity were performed at three points of the model: before the stent-graft implantation site (approximately 2 cm before the graft), within the stent-graft (approximately 2 cm before the model angulation), and within the stent-graft after the angulation (approximately 2 cm distal to the bend). A Toshiba Aplio 400 ultrasound system (Canon Medical Systems, Tokyo, Japan) with a linear ultrasonographic probe was used for this purpose. Each stent-graft implantation was repeated five times in the same angulation model, and the pressures and velocities were recorded (Table 2). The entire procedure was repeated for each tested angulation. All stent-graft deployments were performed under visual guidance by a vascular surgeon with 12 years of experience in EVAR procedures, leveraging the semi-transparency of the printed models. All Doppler ultrasound measurements were performed by a radiologist with 12 years of experience in vascular imaging.

### 2.4. Statistical Analysis

The mean, median, standard deviation (SD), and confidence interval (CI) were used to present continuous variables. The pressure drop across the stent-graft was expressed as the ratio of distal to proximal pressure measurements. To present the increase in flow velocity, the ratio of the values obtained after the kinking to those measured was used. A similar velocity ratio was used to quantify the speed augmentation induced by the stent-graft, defined as the velocity in the proximal segment of the endograft divided by the velocity in the proximal segment of the elastic model. The normality of distribution was verified using the Shapiro–Wilk test, and the homogeneity of variance was assessed using the Levene test. The Kruskal–Wallis test was used for variables with non-normal distributions or unequal variances, while the F-test was used for variables with normal distributions and equal variances. Multiple comparisons were performed using the Kruskal–Wallis post hoc test and the NIR test. A *p*-value < 0.05 was considered statistically significant. All computations were performed using Statistica 13.3 (TIBCO Software Inc., Palo Alto, CA, USA). No formal a priori power analysis was performed, and the study should therefore be considered exploratory in nature.

## 3. Results

### 3.1. Performance at a 75-Degree Angle

At the 75° angulation, the smallest pressure drop was observed for the Anaconda stent-graft (pressure ratio 0.81), whereas lower ratios were recorded for Treo (0.67) and Zenith Spiral-Z (0.63). These differences were statistically significant (*p* = 0.0197 and *p* = 0.0011, respectively) (Table 1).

**Table 1:** Pressure drop ratio for different stent-grafts at angulations of 75°, 90°, and 105°. CI—confidence interval; SD—standard deviation.

Endograft	Angle (°)	Mean	Lower 95% CI	Upper 95% CI	Median	SD
Spiral Z	75	0.62	0.57	0.67	0.64	0.04
Endurant II	75	0.74	0.72	0.75	0.74	0.01
Treo	75	0.66	0.62	0.70	0.68	0.03
Anaconda	75	0.81	0.78	0.83	0.79	0.02
Spiral Z	90	0.69	0.60	0.78	0.68	0.07
Endurant II	90	0.41	0.17	0.65	0.46	0.19
Treo	90	0.69	0.63	0.76	0.68	0.05
Anaconda	90	0.87	0.85	0.89	0.87	0.01
Spiral Z	105	0.62	0.53	0.71	0.60	0.07
Endurant II	105	0.73	0.63	0.83	0.77	0.08
Treo	105	0.66	0.55	0.77	0.68	0.09
Anaconda	105	0.84	0.81	0.86	0.83	0.02

No statistically significant differences in the post-angulation velocity ratio were observed among the tested devices at this angle. Raw pressure and velocity measurements are presented in Table 2.

**Table 2:** Raw pressure and velocity measurements obtained during in vitro flow experiments for each stent-graft and angulation model.

Endograft	Angle (°)	Measurement	Pressure Before Angulation (mmHg)	Pressure After Angulation (mmHg)	Velocity Before Stent-Graft (cm/s)	Velocity in Stent-Graft Before Angulation (cm/s)	Velocity in Stent-Graft After Angulation (cm/s)
Spiral Z	75	1	90	60	100	150	280
Spiral Z	75	2	89	50	90	200	330
Spiral Z	75	3	85	55	100	220	310
Spiral Z	75	4	84	54	85	230	370
Spiral Z	75	5	82	51	90	220	290
Endurant II	75	1	103	78	110	170	250
Endurant II	75	2	98	74	105	150	350
Endurant II	75	3	99	73	105	180	290
Endurant II	75	4	95	69	90	190	430
Endurant II	75	5	89	66	100	200	295
Treo	75	1	95	65	110	180	440
Treo	75	2	102	70	90	200	320
Treo	75	3	87	56	100	190	440
Treo	75	4	101	63	100	170	350
Treo	75	5	93	64	105	220	360
Anaconda	75	1	108	85	120	230	370
Anaconda	75	2	109	87	115	210	340
Anaconda	75	3	107	89	120	230	385
Anaconda	75	4	104	87	115	260	350
Anaconda	75	5	111	88	120	250	350
Spiral Z	90	1	96	77	120	150	300
Spiral Z	90	2	85	55	110	125	300
Spiral Z	90	3	95	65	120	170	420
Spiral Z	90	4	91	66	100	220	300
Spiral Z	90	5	124	76	100	130	450
Endurant II	90	1	125	30	70	200	490
Endurant II	90	2	95	44	80	200	400
Endurant II	90	3	150	30	65	170	430
Endurant II	90	4	92	45	93	170	550
Endurant II	90	5	95	64	110	170	380
Treo	90	1	102	70	100	150	550
Treo	90	2	81	52	110	140	460
Treo	90	3	88	68	120	210	450
Treo	90	4	93	67	120	170	400
Treo	90	5	96	65	80	140	470
Anaconda	90	1	93	83	95	185	260
Anaconda	90	2	99	86	105	250	370
Anaconda	90	3	97	85	110	260	315
Anaconda	90	4	104	90	100	250	340
Anaconda	90	5	103	88	105	210	300
Spiral Z	105	1	96	65	100	150	230
Spiral Z	105	2	96	68	70	190	400
Spiral Z	105	3	103	62	80	200	320
Spiral Z	105	4	96	53	80	220	330
Spiral Z	105	5	100	56	85	240	390
Endurant II	105	1	117	70	105	190	360
Endurant II	105	2	80	64	70	270	320
Endurant II	105	3	91	70	80	150	290
Endurant II	105	4	97	75	90	200	280
Endurant II	105	5	86	62	90	190	320
Treo	105	1	99	50	78	230	600
Treo	105	2	102	70	90	220	410
Treo	105	3	100	68	90	220	480
Treo	105	4	95	67	90	190	540
Treo	105	5	116	84	90	150	370
Anaconda	105	1	99	81	124	230	390
Anaconda	105	2	99	82	130	265	315
Anaconda	105	3	94	81	125	250	320
Anaconda	105	4	101	84	130	240	370
Anaconda	105	5	100	86	125	225	390

### 3.2. Performance at a 90 Degree Angle

At the 90° angulation, the Anaconda stent-graft showed the highest-pressure ratio (0.87), indicating the lowest pressure loss, whereas the Endurant II device showed the lowest ratio (0.41). This difference was statistically significant (*p* = 0.0007). No significant differences were observed among the remaining devices.

Higher increases in post-angulation flow velocity were observed for Zenith Spiral-Z, Endurant II, and Treo compared with Anaconda, reaching statistical significance (*p* = 0.0155, *p* = 0.0064, and *p* = 0.004, respectively) (Table 1).

### 3.3. Performance at a 105-Degree Angle

At 105° angulation, the highest-pressure ratio was again observed with the Anaconda stent-graft (0.84), while lower ratios were observed with Zenith Spiral-Z (0.62) and Treo (0.66). These differences were statistically significant in comparison with the Anaconda device.

Similarly, the increase in the post-angulation velocity ratio was lowest for the Anaconda stent-graft (1.48), whereas the highest value was recorded for the Treo device (2.4), with statistical significance (*p* = 0.0327) (Table 1).

### 3.4. In-Stent Graft Flow Velocity Ratio

The in-stent-graft velocity ratio was defined as the ratio of the velocity in the proximal segment of the stent-graft to the velocity measured in the proximal segment of the elastic model. All tested stent-grafts increased flow velocity within the graft lumen. Mean in-stent velocity ratios were 2.0, 2.1, 1.9, and 2.1 for Zenith Spiral-Z, Endurant II, Treo, and Anaconda, respectively (Table 3). No significant differences were observed among the tested devices.

## 4. Discussion

Stent-graft limb occlusion due to distortion or deformation remains an important reason for secondary intervention after EVAR. Apart from the anatomical factors such as severe angulation or tortuosity of the iliac arteries, reduced vessel diameter due to stenosis with or without calcifications, or a narrow aortic bifurcation, technical aspects of implantation (e.g., excessive oversizing or extension to the external iliac artery) and device-related features may also predispose to iliac limb occlusion [8]. Currently available stent-grafts differ in their stent-strut configurations, including Z-stent, M-stent, O-ring, and spiral designs, which may influence their flexibility and adaptation to complex iliac anatomy, particularly in cases of pronounced arterial angulation [16].

In the present experimental study, we evaluated the hemodynamic performance of four commercially available stent-grafts in compliant 3D-printed iliac bend models with controlled angulations. The obtained results suggest that the device with O-ring-based strut configuration demonstrated more favorable hemodynamic performance under the tested conditions, reflected by higher pressure ratios and lower post-angulation velocity increases compared with other evaluated designs. These findings suggest that stent-strut morphology may influence graft conformability and flow characteristics in angulated vascular segments. However, because the present analysis was limited to pressure and velocity measurements, the mechanisms underlying these differences cannot be fully explained.

The observed results were in line with the instruction for use (IFU) of the Anaconda endograft, which allows implantation in aneurysm neck angulations up to 90°, representing one of the most permissive criteria among currently available devices [17]. In contrast, the IFUs for Endurant II, Treo, and Zenith typically recommended lower angulations [18,19,20]. However, it should be emphasized that IFU criteria primarily refer to proximal neck anatomy, while detailed limits for iliac artery angulation are rarely specified. Moreover, the stent-strut configuration of the main body may differ from that of the iliac limb. For example, the Cook main body employs Z-stents but may be combined with Spiral-Z limbs. Importantly, a substantial proportion of EVAR procedures are performed outside IFU criteria, highlighting the clinical relevance of understanding device behavior in challenging anatomies [21].

Previous experimental and numerical studies also demonstrated that stent-graft design influences mechanical performance in curved vessels. Demanget et al. used finite element analysis to compare commercially available devices and reported that annular stent configurations exhibited greater flexibility and reduced lumen reduction in severe bends compared with Z-stent designs [12]. Similarly, Lin’s group showed that circular or ring-supported stent-grafts were less prone to twisting and fabric damage than Z-stent-supported devices [13].

Clinically, the overall incidence of limb thrombosis after EVAR ranges from approximately 1.4 to 8% and depends on a combination of anatomical and device-related factors. Anatomical risk factors include iliac tortuosity or kinking, severe angulation, small-vessel diameter, or a calcified and narrowed bifurcation [4]. Mechanical graft-related factors, such as twisting, migration, or extrinsic compression, may also contribute to occlusion [14]. The increased pressure loss observed in highly angulated models in the present study may reflect adverse flow conditions. However, its relationship to in vivo thrombosis cannot be determined in this in vitro setup. A systematic review by Coelho et al. reported that endograft limb kinking accounted for 42.8% of limb occlusion cases, suggesting that geometric deformation plays a major role in limb failure [22].

Several clinical studies further indicate that device design may influence limb patency. Shintani et al. reported lower occlusion rates in patients treated with helical stent-supported limbs on the external iliac artery [15]. Additional placement of a bare-metal stent has also been suggested as a protective measure in cases at high risk of limb compression or kinking [23]. Marques de Marino’s group similarly observed higher occlusion rates with Z-stent-supported designs compared with more flexible designs, and registry-based analyses have shown variability in limb occlusion rates across devices [24,25]. Although the present in vitro results cannot be directly translated into clinical outcomes, they suggest that device flexibility and conformability may influence limb behavior in angulated anatomy under controlled experimental conditions.

This study has several limitations that should be acknowledged. First, the experiments were performed in simplified compliant arterial phantoms with a single controlled angulation rather than full patient-specific iliac geometries. The models did not include the stent-graft main body or aortic bifurcation, which may influence limb positioning and mechanical interactions in vivo. Previous computational and imaging-based studies have shown that patient-specific anatomy, device configuration, and boundary conditions may substantially influence predicted stent-graft behavior and local hemodynamic parameters [26,27]. Therefore, the present setup should be interpreted as a partial model of iliac limb behavior under standardized geometric conditions rather than as a realistic EVAR reconstruction or direct simulation of clinical anatomy. This is particularly important because post-EVAR thrombotic complications have been linked to both aorto-iliac geometry and local hemodynamic disturbances in patient-specific analyses [27,28]. Accordingly, the present findings cannot be directly extrapolated to clinical limb occlusion risk. Second, only iliac limbs with diameters of 12–13 mm were tested, reflecting commonly used clinical sizes, but not covering the full range of available diameters. In addition, the Anaconda device was tested with a 12 mm limb, whereas the other stent-grafts were tested with 13 mm limbs, because no 13 mm Anaconda option was available. The nominal limb lengths also differed between devices (80 mm for Anaconda, 82 mm for Endurant II, 80 mm for Treo, and 90 mm for Zenith Spiral-Z). These differences in diameter represent a potential confounding factor and should be considered when interpreting the observed hemodynamic differences, as the results cannot be attributed solely to stent-strut morphology. Device sizing and oversizing may influence endograft behavior and hemodynamics, warranting further investigation. Third, although the compliant material used for printing approximates arterial elasticity, it does not fully reproduce the mechanical properties of diseased human vessels, particularly in the presence of calcifications or stenosis. Fourth, perfusion was performed using a water-glycerol mixture representing a Newtonian fluid, which does not fully replicate the non-Newtonian rheological properties of blood and may affect detailed flow patterns. Fifth, the present analysis was restricted to pressure and flow velocity measurements and did not include wall shear stress, detailed flow pattern assessment, or lumen deformation analysis, which limits mechanistic interpretation of the observed differences between stent-graft designs. Sixth, each experimental setting was repeated five times, and no formal power analysis was performed. Therefore, the statistical comparisons should be interpreted with caution, particularly in the context of multiple comparisons across devices and angulations. Finally, long-term geometric changes in implanted limbs, such as increased curvature or reduced inter-ring distance, reported in follow-up studies of Anaconda devices, were not addressed in this short-term experimental setup [29].

Despite these limitations, the controlled in vitro design allowed direct comparison of different stent-strut configurations under identical flow and geometric conditions, providing experimental insight into the relationship between graft structure and global hemodynamic behavior in angulated iliac segments. These findings may provide experimental insights into device behavior in challenging iliac anatomy and should be considered hypothesis-generating bench data for future clinical studies.

## 5. Conclusions

The results of this experimental study suggest that stent-grafts with an O-ring-based strut configuration may provide more favorable hemodynamic performance in severely angulated iliac segments under controlled in vitro conditions. This design demonstrated higher pressure ratios and lower post-angulation velocity increases compared with other tested configurations, indicating improved conformability in the evaluated models.

These findings suggest that stent-graft structural design may influence device behavior in relation to vessel geometry under controlled in vitro conditions. These observations should be interpreted as hypothesis-generating bench data and may be relevant for future investigations of EVAR in angulated iliac anatomy.

Furthermore, compliant 3D-printed vascular phantoms may serve as a useful experimental tool for evaluating stent-graft behavior under challenging anatomical conditions and for supporting future device optimization. Additional studies incorporating more complex vascular geometries, full aorto-iliac reconstructions, advanced hemodynamic analyses such as wall shear stress and flow pattern assessment, and clinical and longitudinal outcomes such as limb patency and thrombosis are required to determine the translational relevance of these findings.

## Figures and Tables

**Table 3:** Post-angulation flow velocity ratio for different stent-grafts at angulations of 75°, 90°, and 105°. CI—confidence interval; SD—standard deviation.

Endograft	Angle (°)	Mean	Lower 95% CI	Upper 95% CI	Median	SD
Spiral Z	75	1.57	1.3	1.83	1.61	0.22
Endurant II	75	1.83	1.29	2.37	1.61	0.43
Treo	75	2.01	1.53	2.49	2.06	0.38
Anaconda	75	1.52	1.35	1.71	1.61	0.14
Spiral Z	90	2.34	1.39	3.29	2.40	0.76
Endurant II	90	2.49	1.91	3.07	2.45	0.46
Treo	90	2.96	2.12	3.79	3.29	0.67
Anaconda	90	1.37	1.25	1.50	1.40	0.10
Spiral Z	105	1.67	1.36	1.98	1.60	0.25
Endurant II	105	1.62	1.22	2.02	1.68	0.32
Treo	105	2.39	1.92	2.86	2.47	0.38
Anaconda	105	1.49	1.18	1.79	1.54	0.24

## Data Availability

The datasets analyzed during the current study are available from the corresponding author upon reasonable request.

## Abbreviations

The following abbreviations are used in this manuscript:

3D	Three-Dimensional
AAA	Abdominal Aortic Aneurysm
CIA	Common Iliac Artery
CI	Confidence Interval
CTA	Computed Tomography Angiography
EIA	External Iliac Artery
EVAR	Endovascular Aneurysm Repair
SD	Standard Deviation
